# Oral bioaccessibility and probabilistic human health risk assessment of potentially toxic elements in stream sediments from an abandoned gold mine in Panama

**DOI:** 10.1007/s10653-025-02535-4

**Published:** 2025-05-23

**Authors:** Ana Cristina González-Valoys, Samantha Jiménez-Oyola, Carla Patinha, Eva Ma García-Noguero, Jesús Peco, Felipe Segundo, José Ignacio Barquero, Miguel Vargas-Lombardo, José María Esbrí, Pablo Higueras

**Affiliations:** 1 Facultad de Ingeniería Civil, Universidad Tecnológica de Panamá, Ricardo J. Alfaro Avenue, Dr. Víctor Levi Sasso University Campus, Panamá City, 0819-07289 Panama; 2 SNI-SENACYT Sistema Nacional de Investigación-Secretaria Nacional de Ciencia, Tecnología e Innovación, Clayton, Ciudad del Saber Edif.205, Panamá City, 0816-02852 Panama; 3 Centro de Estudios Multidisciplinarios en Ciencia, Ingeniería y Tecnología (CEMCIT-AIP), Ricardo J. Alfaro Avenue, Dr. Víctor Levi Sasso University Campus, Panamá City, 0819-07289 Panama; 4 Escuela Superior Politécnica del Litoral, ESPOL, Facultad de Ingeniería en Ciencias de la Tierra, Campus Gustavo Galindo Km 30.5 Vía Perimetral, P.O. Box 09-01-5863, Guayaquil, Ecuador; 5 GeoBioTec, GeoBioSciences, GeoTechnologies and GeoEngineering Research Center and Department of Geosciences, University of Aveiro, Campus de Santiago, 3810-193 Aveiro, Portugal; 6 Instituto de Educación Secundaria (IES) Mercurio, 13400 Almadén, Ciudad Real Spain; 7 Dpto. Producción Vegetal y Tecnología Agraria, ETSIA, Universidad de Castilla-La Mancha, Ciudad Real, Spain Ronda de Calatrava, 13003; 8 Instituto de Geología Aplicada, Universidad de Castilla-La Mancha, EIMI Almadén, Plaza Manuel Meca 1, 13400 Almadén, Ciudad Real Spain; 9 Facultad de Ingeniería de Sistemas Computacionales, Universidad Tecnológica de Panamá, Ricardo J. Alfaro Avenue, Dr. Víctor Levi Sasso University Campus, Panamá City, 0819-07289 Panama; 10 Departamento de Mineralogía y Petrología, Universidad Complutense de Madrid, José Antonio Novais 12, 28040 Madrid, Spain

**Keywords:** Stream sediments, In-vitro bioaccessibility, Probabilistic assessment, Gold mining

## Abstract

In this study, the oral bioaccessibility of potentially toxic elements (PTEs) (As, Ba, Cu, Sb, and Zn) in river sediments from the abandoned Remance Mine was evaluated, and the associated human health risks from exposure to these contaminants through accidental ingestion during recreational activities were assessed using a probabilistic approach. The pseudo-total concentrations and bioaccessible fractions (BAF) of the selected PTEs were determined using the Unified BARGE Method (UBM), which simulates the human digestive process for both the gastric (G-phase) and gastrointestinal (GI-phase) phases. The results indicate that the BAF of PTEs was higher in the G-phase than in the GI-phase. In the G-phase, the BAF followed this decreasing order: Cu > Ba > Zn > As > Sb, while in the GI-phase, the order was Cu > Zn > Ba > As > Sb. Regarding the risk assessment, As emerged as the most significant contaminant, exceeding the safe exposure limits for both carcinogenic (CR) and non-carcinogenic (HQ) risk, mainly in children. The CR for the pseudo-total concentration was 10 times higher than in the G-phase and 18 times higher than in the GI-phase. The HQ results indicated values exceeding the safe exposure threshold only in the pseudo-total concentration. These findings highlight that the incorporation of bioaccessibility into risk assessments provides more accurate estimates. This is a novel study, the first one carried out in Panama, which investigates the oral bioaccessibility of potentially toxic elements (PTEs) in stream sediments from the Remance gold mine, an area with high concentrations of As, Cu, Zn, Sb, and Ba. Finally, the importance of managing river use in contaminated mining environments is underscored, and some recommendations are provided, aimed to make these sustainable.

**Supplementary Information:**

The online version contains supplementary material available at 10.1007/s10653-025-02535-4.

## Introduction

Mining activity, particularly gold mining, represents a significant source of environmental pollution due to the large amount of waste it generates, among which mining tailings play a crucial role. These wastes contain potentially toxic elements (PTEs) such as As, Cd, Pb, Sb, and Zn, which can persist in the environment and bioaccumulate in human organisms, posing serious risks to both human health and ecosystems (Dintsi et al., 2023; Lima et al., 2022; Mehta et al., 2020; Xie et al., 2023). Specifically, these mining residues are often abandoned without adequate closure measures, leaving large volumes of tailings and waste exposed to erosion and runoff (Garcia-Ordiales et al., 2018; Guzmán-Martínez et al., 2022; Rouhani et al., 2022). When these mine residues are incorporated into rivers, the contaminants contained in them can remain suspended in the water or settle in the riverbed sediments. Turning these sediments into reservoirs for PTEs and a primary source of exposure for aquatic ecosystems and, potentially, for nearby human communities (Beck et al., 2020; Arranz-González et al., 2021; García-Martínez, et al., 2021; Jiménez-Oyola et al., 2023). Beyond Panama, abandoned mines and insufficient remediation efforts are common worldwide, and therefore the findings of this research may serve as a model for other regions dealing with similar legacy mining issues.

Exposure to contaminated sediments can mainly occur through accidental ingestion or dermal contact during recreational activities in the rivers, which represents a public health threat. Recreational activities in contaminated water bodies are common in warm climates and developing countries. Several studies have documented non-carcinogenic and carcinogenic risks associated with exposure to contaminated sediments in mining areas, highlighting that accidental ingestion is the exposure pathway that contributes most to these risks (Jiménez-Oyola et al., 2021a, 2021b).

Traditionally, the total concentration of PTEs in sediments is considered in health risk assessments. However, it is essential to understand that not all the PTEs fraction present in sediments is bioavailable for absorption by the human body (O’Connor et al., 2021). Bioavailability refers to the amount of contaminant that, when ingested, inhaled, or absorbed through the skin, reaches the circulatory system and human organs (Bourliva et al., 2021; Kastury et al., 2024). Instead of relying solely on total PTE levels, measuring the bioavailable fraction provides a more accurate picture of potential health risks (Lyu et al., 2020; Sun et al., 2024).

In the mining area of Remance, Panama, González-Valoys et al., (2021) reported high concentrations of PTEs in river sediments, as well as a potential risk to the ecosystem and human health. However, risk assessments of the study were carried out based on the total concentrations of PTEs and using a deterministic methodology that can overestimate or underestimate the real risks by ignoring variations in exposure parameters. Therefore, it is crucial to investigate the bioaccessibility of PTEs present in Remance sediments, as only a fraction of these contaminants is solubilized and absorbed by the human body. In vitro bioaccessibility tests, which simulate human gastrointestinal conditions, have shown good correlation with in vivo animal tests, allowing for a more realistic estimation of human exposure levels (Corona Sánchez et al., 2021; Soltani et al., 2021).

One of the methods to estimate oral bioaccessibility is the Unified BARGE method (UBM), developed by the Bioaccessibility Research Group of Europe (BARGE), in which the fraction of the contaminant that is solubilized in the human gastrointestinal tract and available to be absorbed is determined (Wragg et al., 2011). This method has been validated for in vivo studies for As, Cd and Pb (Denys et al., 2009, 2012), and it is widely used for the assessment of risks to human health produced by abandoned mine sites (Mehta et al., 2020; Soltani et al., 2021; Xie et al., 2023).

Furthermore, traditional deterministic methodology, although useful as a first step in risk assessment, does not account for the uncertainty and variability inherent in exposure (Mohammadpour et al., 2023).Therefore, probabilistic risk analysis has become a more suitable approach, as it allows the integration of variables that describe the probability distribution of an event, providing a more precise estimate of the percentage of the population exposed to health risks from a specific contaminant (Jiménez-Oyola, Escobar Segovia, et al., 2021a, 2021b; USEPA, 2011). In addition to its scientific value, this research can guide practical policies in areas affected by mining. For instance, local authorities could use these findings to regulate recreational water activities, organize sediment clean-up efforts, or launch public health campaigns aimed to prevent accidental ingestion of contaminated materials.

PTEs (As, Ba, Cu, Sb, and Zn) were identified in a previous study as elements that represented potential ecological and human health risks through accidental ingestion (González-Valoys et al., 2021, 2022). Stream sediments are the most affected by the mining activity, as tailings flow into streams and release material through runoff, being Panama a tropical country with high rainfall (González-Valoys et al., 2021).

In this context, the aim of this study is to evaluate the oral bioaccessibility of PTEs (As, Ba, Cu, Sb, and Zn) in river sediments from the Remance Mine, Panama, and estimate the probabilistic risk associated with human exposure to these contaminants. This approach will allow a more accurate assessment of the health risks derived from recreational activities in water bodies, providing essential data for environmental management and the protection of vulnerable populations worldwide.

## Materials and methods

### Study area

The Remance gold mine is in a district of San Francisco, Veraguas Province, Panama. The gold mineralization occurs as small inclusions within pyrite and marcasite, as well as free gold, disseminated within quartz veins, together with other accessory minerals in small quantities such as chalcopyrite (CuFeS_2_), sphalerite (ZnS), galena (PbS) and arsenopyrite (FeAsS). Also, Ag and As are found along with anomalous amounts of Sb and locally Hg (Nelson & Ganoza, 1999). The geology of the site corresponds to volcanic rocks of the type dacites, rhyodacites, rhyolites, tuffs, agglomerates, basalts and andesites (STRI, 2021). According to mineralogical analysis, the stream sediment samples have a composition of kaolinite (5–10%), illite (< 5%), chlorite (< 25%), quartz (65–100%) and feldspar (< 10%) (González-Valoys et al., 2021).

The mine had its last period of activity between 1989 and 1999 (Nelson & Ganoza, 1999), ceasing its exploitation activities without an adequate mine closure process. Exposed to the environmental conditions, three tailing ponds were left with fine, cyanide-processed waste materials, as well as an area to the south of the mining concession where an open pit excavation took place on Tullido hill. The unprocessed wastes from this area are evident sources of PTEs, including As, Ba, Cu, Sb and Zn (González-Valoys et al., 2021), which have been disseminated by runoff to the stream sediments, resulting in these being affected with a load of considerable contamination, posing a potential risk to the health of ecosystems and river users.

The climate of the area corresponds to the AMI type according to the Koppen classification (IMHPA, 2021). This is a humid tropical climate, influenced by the monsoon, with annual rainfall > 2250 mm concentrated in the months of August to November, and the driest months from January to March, with an average annual temperature > 18 °C (IMPHA, 2007).

### Sampling collection

**Fig. 1:**
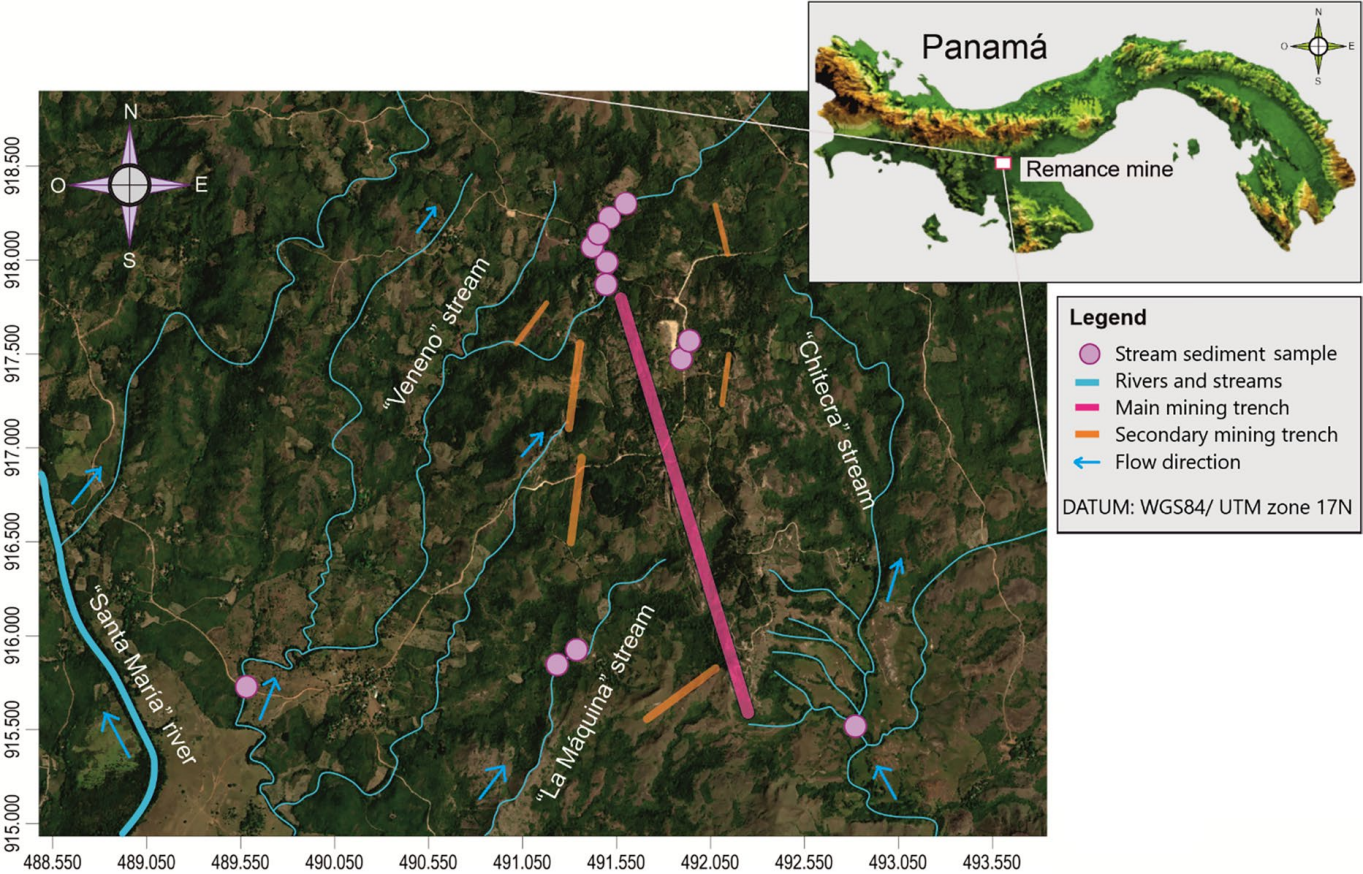
Sampling station of stream sediment from the Remance mine, Panama

Surface bottom sediment samples (0–10 cm depth) were collected from twelve sampling points in streams at different locations near areas of mining activity (Fig. 1). We selected this sampling depth based on standard sediment sampling protocols for assessing surface contamination, where the top 10 cm is most relevant for human exposure (Simpson, 2005). These sampling points were chosen to study the influence of anthropogenic pollution sources on sediment quality, as well as to evaluate the bioaccessibility for selected PTEs (As, Ba, Cu, Sb, and Zn) in the environment, based on a previous study in the area where these elements were identified as having potential ecological and human health risks through accidental ingestion (González-Valoys et al., 2021). Sediment samples were collected during the rainy season, because it is the period of the annual cycle in which the expected mobility of PTEs is maximum, thus representing the worst-case scenario (Rajeshkumar et al., 2018). Approximately 3 kg of sample was taken with a plastic shovel and stored in plastic bags. Samples were taken to the laboratory and immediately air-dried for one week (González-Valoys et al., 2021) to avoid changes in composition due to oxidation or reduction.

### Sample preparation and physical–chemical analysis

All the sediment samples were dried at room temperature, disaggregated and passed through a 2 mm sieve. An aliquot of the < 2 mm fraction was passed through a 250 µm sieve to determine the digestible fractions, including the pseudo-total concentration and the oral bioaccessibility test.

Edaphic parameters were determined in the < 2 mm fraction. pH, electrical conductivity (EC), oxide reduction potential (ORP) were determined in a 1:5 (w/v) suspension (ASTM, 2019) using an Orion Versa Star Pro benchtop multiparameter device. Organic matter (OM) was quantified by weight loss at 440 °C (ASTM, 2020). Cation exchange capacity (CEC) was determined by the potentiometer method (Weaver et al., 1991). Soil texture classification was determined by the Bouyoucos method (ASTM, 2021).

#### Elemental extraction and ICP-MS analysis

Pseudo-total concentration is used in risk assessment studies for heavy metals in soils and sediments as it estimates the bioavailable fraction of metals (Gupta et al., 1996). The pseudototal concentration is estimated through an acid digestion that dissolves the metals not bound or weakly bound to the silicate phases, which will be the bioavailable ones (Hödrejärv & Vaarmann, 1999).

The acidic digestion of the samples consisted in the addition of 3 mL of concentrated nitric acid (HNO_3_) and 1 mL of hydrochloric acid. After overnight digestion (14–16 h), the solutions were placed in a digestion block (DigiPrep, SCP Science; Montreal, QC, Canada) and two heating cycles were performed. These consisted in the increase, for 10 min, from room temperature to 50 °C, which was kept stable during a period of 15 min. The temperature then increased, in 15 min, from 50 to 85 °C and stabilized for another 15 min. After digestion, samples were diluted with Millipore water (Milli–Q) to a final concentration of 1–2% HNO_3_, to reduce acid concentration and avoid damaging the inductively coupled plasma mass spectrometry (ICP-MS) equipment.

The pseudo-total concentration of PTEs was determined using an Agilent 7700 ICP-MS (Agilent Technologies, Santa Clara, CA, USA) equipped with an octopole reactions system (ORS) collision/reaction cell technology to minimize spectral interferences. Germanium (72Ge), rhodium (103Rh), and terbium (193 Tb) were used as internal standards. For quality assurance and control (QA/QC), certified reference materials TILL 1 (Canadian Certified Reference Materials), reagent blanks, and analytical duplicates were also digested. Results recorded for procedural blanks were always below the limit of detection and mean recoveries for the elements referred above ranged from 90 to 100%, with relative standard deviation (RSDs) for all replicates was < 10%.

#### Oral bioaccessibility extraction

**Fig. 2:**
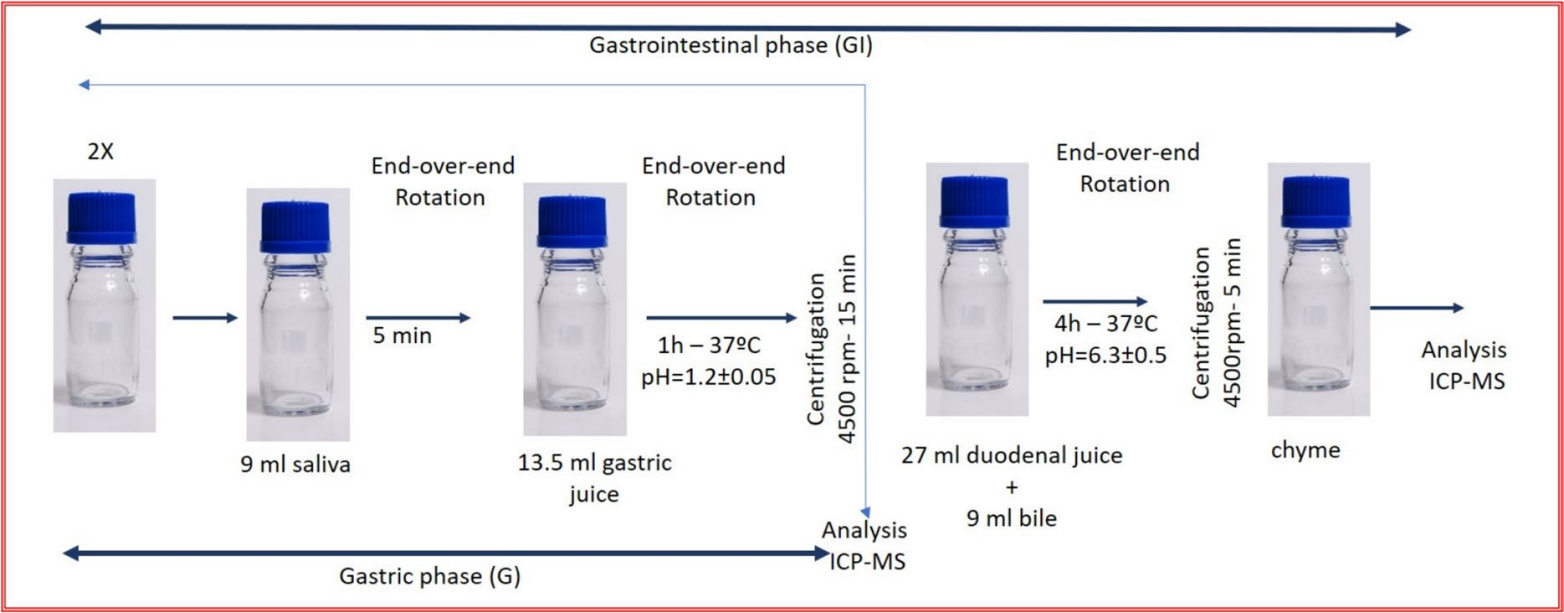
Schematic representation of the UBM method procedure

The Unified Bioaccessibility Method (UBM) for in vitro bio accessibility testing was applied to the sediment samples and determined for As, Ba, Cu, Sb, and Zn. The oral bioaccesibility test was performed at GeoBioTec Research Unit Laboratory, University of Aveiro, Portugal. Oral bioaccessibility was performed in two-stage in vitro procedure (Fig. 2), which mimics the human digestive procedure, simulated digestive fluids, representing saliva and gastric juice for the Gastric phase (G-phase), plus bile and duodenal juice for the Gastrointestinal phase (GI-phase). The chemical constituents included in each phase were the same as those previously reported (Oomen et al., 2002; Wragg et al., 2011); they were produced from inorganic and organic reagents and used to replicate the three main compartments of the human gastro-intestinal tract involved in digestion: mouth, stomach and small intestine (Bourliva et al., 2021; Wragg et al., 2011). The UBM-extracted contents were analyzed using an inductively coupled plasma mass spectrometer (ICP-MS). For quality assurance blanks, duplicate samples, and the UBM-specific certified reference material BGS 102 (Hamilton et al., 2015) were used to validate the quality of the extraction protocols. The recovery percentage for the reference material BGS 102 was 100% in the gastric and gastrointestinal phase for As, Ba, Cu, Sb, and Zn, according to the concentrations of Hamilton et al. (2015), with relative standard deviation (RSDs) for all replicates was < 10%.

The Bioaccessible Fraction (% BAF) was calculated as the ratio between the bioaccessible fraction and the pseudo-total concentration (Eq. 1) (Bourliva et al., 2021); where bioaccessible fraction of element is bioaccessibility concentration in both G and GI phases, separately.

1 $$\% {\text{ BAF}}\, = \,\frac{{Bioaccessible \,fraction\, of\,element\, \left( {\frac{{{\text{mg}}}}{{{\text{kg}}}}} \right)}}{{Pseudo\,total \,fraction \,of\, element \left( {\frac{{{\text{mg}}}}{{{\text{kg}}}}} \right)}} \times 100$$

### Exposure assessment and risk characterization

The purpose of a risk assessment for carcinogenic and non-carcinogenic effects is to estimate the potential for PTEs to cause harmful effects in exposed people, especially children. For this purpose, the human health risk was evaluated considering a recreational scenario where recipients, adults and children, are exposed to PTEs through the accidental ingestion of stream sediments during recreational activities in local rivers.

The UBM methodology evaluates the gastric and gastrointestinal phases separately due to the difference in pH and body fluids involved. In particular, at acid pH in the stomach (1.5–2.0 pH) in the gastric phase some elements are more bioaccessible (H. B. Li et al., 2014; Pelfrêne & Douay, 2018), while in the gastrointestinal phase at a more neutral pH (5.5–7.0 pH) other elements are more bioaccessible (Mehta et al., 2020).

The average daily dose (ADD: mg kg^−1^ day^−1^) for accidental ingestion of sediments was calculated for the three fractions: pseudo-total concentration, gastric and gastrointestinal phases, for both receptors, using Eq. 2 proposed by USEPA, (2014).

2 $$ADD_{oral} { } = { }\frac{{C_{i} \times {\text{ EF }} \times {\text{ ED }} \times {\text{ IR}}}}{{\text{AT x BW}}} \times {\text{ CF}}$$

where C*i* is the concentration of each element in stream sediment (in mg kg^−1^), EF is the exposure frequency (day year^−1^), ED is the life exposure duration (years), IR is the ingestion rate (mg kg^−1^), AT is the averaging time (ED x EF day year^−1^ in non-carcinogenic risk and 70 × EF day year^−1^ in carcinogenic risk), BW is the body weight (kg), and CF is the conversion factor (1 × 10^–6^).

The Non-Carcinogenic Risk was calculated with the hazard quotient (HQ) as follows in Eq. 3. The cumulative non-cancer risk was determined using the hazard index (HI), as the sum of the hazard quotients for the 1–n PTEs investigated (Eq. 4). The RfD is the reference dose (mg kg^−1^ day^−1^), which is the daily input that allows an individual to maintain that level of exposure over a long period of time without experiencing any adverse effects to human health. This parameter must be independently derived from compound-specific epidemiological or laboratory data and is used to estimate non-carcinogenic health risks (Chattopadhyay, 2005). For the study elements are: RfD_As_ = 3.0 × 10^–4^, RfD_Ba_ = 2.0 × 10^–1^, RfD_Cu_ = 4.0 × 10^–2^, RfD_Sb_ = 4.0 × 10^–4^, and RfD_Zn_ = 3.0 × 10^–1^.

The carcinogenic risk (CR) risks were calculated as follows in Eq. 5, where SF is the slope factor for oral over a lifetime for a particular PTEs that plays a key role in daily toxin intake and results in an increased risk of an individual developing cancer. Only As was considered in the carcinogenic risk assessment, as the other PTEs have no known carcinogenic effects. The SF for As is 1.5. Toxicity values RfD and SF were taken from the The Risk Assessment Information System website (USDoE, 2024).

3 $${\text{HQ}} = \frac{{{\text{ADD}}}}{{{\text{RfD}}}}$$

4 $${\text{HI }} = { }HQ_{1} { } + { }HQ_{2} { }.{ }.{ }.{ } + {\text{ HQ}}_{n}$$

5 $${\text{CR}} = {\text{ADD}} \times {\text{SF}}$$

#### Probabilistic risk assessment

In this study, the probabilistic risks to human health from accidental sediment ingestion in a recreational setting were calculated for the three fractions (pseudo-total concentration, gastric, and gastrointestinal phases), for both carcinogenic and non-carcinogenic risk for children and adults. The probabilistic risk assessment involves using the probability distributions of the input parameters in the risk equation, resulting in a distribution of risk values (Mendoza et al., 2023; Mohammadpour et al., 2023).

The resampling technique was applied to the PTEs concentrations for the pseudo-total concentration, gastric, and gastrointestinal phases; this data resampling was done with R software. The data generated (*n* = 1000 for each PTE) was used as a probability distribution in the risk equations to calculate the probabilistic risk for accidental sediment ingestion. The exposure parameters used for the probabilistic risk assessment correspond to: EF (days year^−1^) for adults and children with triangular distribution 120 (26–260); BW (kg) for adults with normal distribution 72 ± 15.9, and BW (kg) for children with normal distribution 15.6 ± 3.7 (Jiménez-Oyola et al., 2021a, 2021b). For ED and IR, a point estimated value was used: ED is 30 years in an adult and 6 years in a child (Israeli & Nelson, 1992; Spence & Walden, 2001) and IR is 12.5 mg kg^−1^ in an adult and 50 mg kg^−1^ in a child (Goldblum et al., 2006). The equations used to calculate the ADD, HQ, HI and CR were implemented in R programming language (R Core Team, 2020).

### Data processing

Descriptive statistics were used to observe the trends in the data set. In addition, a multifactorial analysis was carried out to observe the behavior of the study variables. The data were statistically analyzed using Microsoft 365 (office) Excel, Minitab version 15, and R programming language. The Surfer program (version 25) was used to create maps.

## Results

### Physicochemical characteristics of the stream sediments

The physicochemical characteristics of stream sediments samples in the < 2 mm fractions are given in Table 1. The soil pH ranged between 3.0 and 6.0, being between acidic to slightly acidic. The OM in the stream sediments at p50 is 3.3%, evidencing the degree of erosion (Li et al., 2019). High amounts of As, and other elements such as Cu, Sb and Zn linked to the activity of gold mining extraction (Chen et al., 2023) in the area are observed. Regarding texture with the samples mostly having a sandy and sandy loam texture which are associated with the presence of PTEs in mining work areas (Achour et al., 2022; Kowalkowski et al., 2010).

**Table 1:** Statistical summary of the physicochemical parameters and PTEs concentration analyzed in stream sediments (< 2 mm fraction)

Parameter	Min	p50	p95	Max	SD
pH	3.0	4.9	5.9	6.0	0.9
EC (dS m^−1^)	0.023	0.098	0.572	1.024	0.273
ORP (mV)	114.1	507.0	686.0	738.0	180.8
OM (%)	0.9	3.3	9.1	9.5	2.9
CEC (cmol kg^−1^)	5.7	9.6	13.2	13.5	2.6
As (mg kg^−1^)	95.7	226.5	693.4	714.5	211.3
Ba (mg kg^−1^)	142.7	444.0	537.5	549.0	146.8
Cu (mg kg^−1^)	26.8	82.6	134.6	136.2	45.7
Sb (mg kg^−1^)	15.5	29.6	48.1	55.9	12.1
Zn (mg kg^−1^)	18.5	56.2	144.8	166.6	42.6
Texture—Clay (%)	0.7	8.3	32.4	35.1	11.4
Texture—Silt (%)	4.7	17.2	36.4	36.7	12.3
Texture—Sand (%)	33.1	73.5	92.4	92.4	22.2

*EC* electrical conductivity, *ORP* oxidation–reduction potential, *OM* organic matter, *CEC* cationic exchange capacity

### Pseudo-total and bioaccessible concentrations of PTEs

**Fig. 3:**
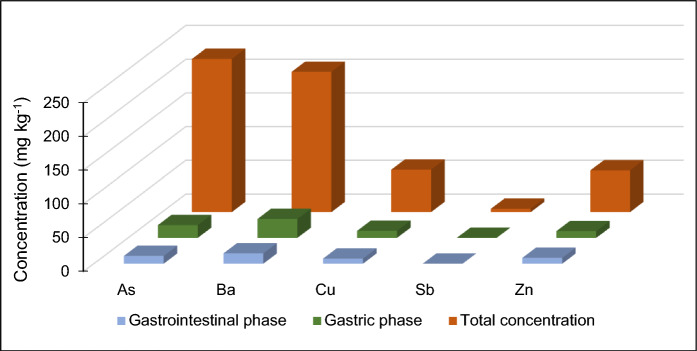
Pseudo-total concentration and the gastro and gastrointestinal phase for the concentration (p50) of selected PTEs of the in vitro test

Figure 3 shows the bar graph for the PTE_S_ (As, Ba, Cu, Sb, and Zn), including the pseudo-total concentrations and the bioaccessible fractions for the in vitro gastro and gastrointestinal phases analyzed in the stream sediments (< 250 µm fraction). Table S1 comprises the statistical summary of pseudo-total contents of PTEs, and data on bioaccessible levels in gastric and gastro-intestinal phases as well as calculated bioaccessible fractions (BAF).

Regarding the pseudo-total contents of the sediments in the Remance streams, PTEs concentration (p50) was found in the following decreasing order: As (227.2 mg kg^−1^) > Ba (208.1 mg kg^−1^) > Cu (62.7 mg kg^−1^) > Zn (61.7 mg kg^−1^) > Sb (4.9 mg kg^−1^). Regarding the bioaccessible concentrations in the gastro phase at a pH of 1.5 we have the following decreasing order: Ba (28.2 mg kg^−1^) > As (19.0 mg kg^−1^) > Cu (10.5 mg kg^−1^) = Zn (10.2 mg kg^−1^) > Sb (0.1 mg kg^−1^). For the gastrointestinal phase at a pH of 7.0, the decreasing order was as follows: Ba (15.4 mg kg^−1^) > As (11.5 mg kg^−1^) > Zn (8.7 mg kg^−1^) > Cu (7.6 mg kg^−1^) > Sb (0.1 mg kg^−1^).

**Fig. 4:**
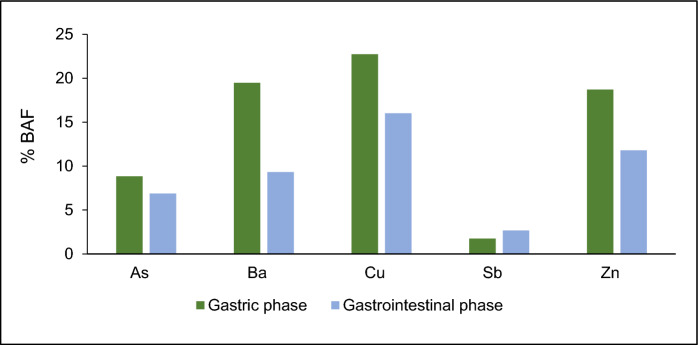
Oral bioaccessible factor (BAF%) for the concentration (p50) of selected PTEs in gastric and gastrointestinal phases

Figure 4 shows the bar graph for the oral bioaccessible factor (BAF%) of the concentration (p50) of selected PTEs in gastric and gastrointestinal phases. The decreasing order of the BAF in the gastric phase was Cu (22.7%) > Ba (19.5%) = Zn (18.7%) > As (8.8%) > Sb (1.7%). The decreasing order of the BAF in the gastrointestinal phase was Cu (16.9%) > Zn (12.8%) > Ba (9.5%) > As (7.5%) > Sb (2.7%). The general trend of the PTEs was to decrease their bioavailability in the gastrointestinal phase where the process occurs at neutral pH. Cu shows the highest bioaccumulation in both phases (gastric and gastrointestinal). These results indicate that Cu is more easily absorbed and retained in the organism compared to other PTEs. Cu is an essential trace element in the human body, but in high concentrations, it can be toxic. Regarding Zn, another essential element, it has considerable BAF in both phases (gastric 18.7% and gastrointestinal 12.8%), so although it has a crucial biological function, an excess of Zn can also be harmful. Ba shows a significant accumulation pattern in the gastric phase (19.5%), however, the BAF decreases by about 50% in the gastrointestinal phase. Ba is less toxic compared to other PTEs, but its accumulation could lead to long-term health effects if exposure is constant. Finally, As and Sb are the PTEs that show the least bioaccumulation compared to the other PTEs. As, although highly toxic, shows a low BAF in both phases (8.8% in the gastric phase and 7.5% in the gastrointestinal phase), suggesting that its absorption in the body is limited. On the other hand, Sb, with the lowest BAF values (1.7% in the gastric phase and 2.7% in the gastrointestinal phase), suggests that it accumulates less efficiently in the organism. Although As and Sb show the lowest BAF in this study, this does not necessarily reflect an absence of risks if present at high concentrations in the environment.

The BAF responds to the solubility behavior of the elements according to pH and body solutions, with Ba, Cu and Zn being more bioaccessible at the acid pH (Bravo et al., 2017) of the gastric phase, while in the gastrointestinal phase, where pH is neutral, their bioaccessibility decreases. The opposite is true for Sb, which is more bioaccessible at less acid pH (Bolan et al., 2022) in the gastrointestinal phase.

The BAF for As (1.5–17.2% in the G-phase and from 1.7 to 13.6% in the GI-phase) (Table S1) are within the range of BAFs reported by Gaberšek and Gosar, (2024) who evaluated the bioavailability of several PTEs in urban environmental soil (As—BAF% between 6.8–27.9% in G-phase and 5.84–16.3% in GI-phase). In contrast, Das et al. (2013) evaluated the bioaccessibility of As in soils using the simplified bioaccessibility extraction test and obtained values ranging from 5.7 to 46.3%. Regarding the bioaccessibility of the other PTEs, the results of our studies differ from those reported by Gaberšek and Gosar (2024): Cu (BAF% between 23.5% to 54.9% in G-phase and between 25.4 and 53% in GI-phase), Sb (BAF% between 0.26 and 1.48% in G-phase and − 0.3% in GI-phase), and Zn (BAF% between 20.8–70.7% in G-phase and 0.53–5.69% in GI-phase). This variability is due to a combination of factors such as the spatial distribution of PTEs, chemical speciation, solubility, type of materials, climatic conditions, among others (Kastury et al., 2024; O’Connor et al., 2021).

### Analysis of factors related to oral bioaccessibility

Other factors, such as the physicochemical properties of the material, can influence oral bioaccessibility in the gastric and gastrointestinal phases, including PETs total concentration, OM, pH, and grain size (sand, silt, clay) (Soltani et al., 2021). The presence of PTEs could be associated with clay fractions, such as kaolinite and illite (Palansooriya et al., 2020). This multiparametric possible influences makes it necessary to carry out a multifactorial analysis to observe which variables may be influencing the oral bioaccesibility to a greater or lesser extent.

**Fig. 5:**
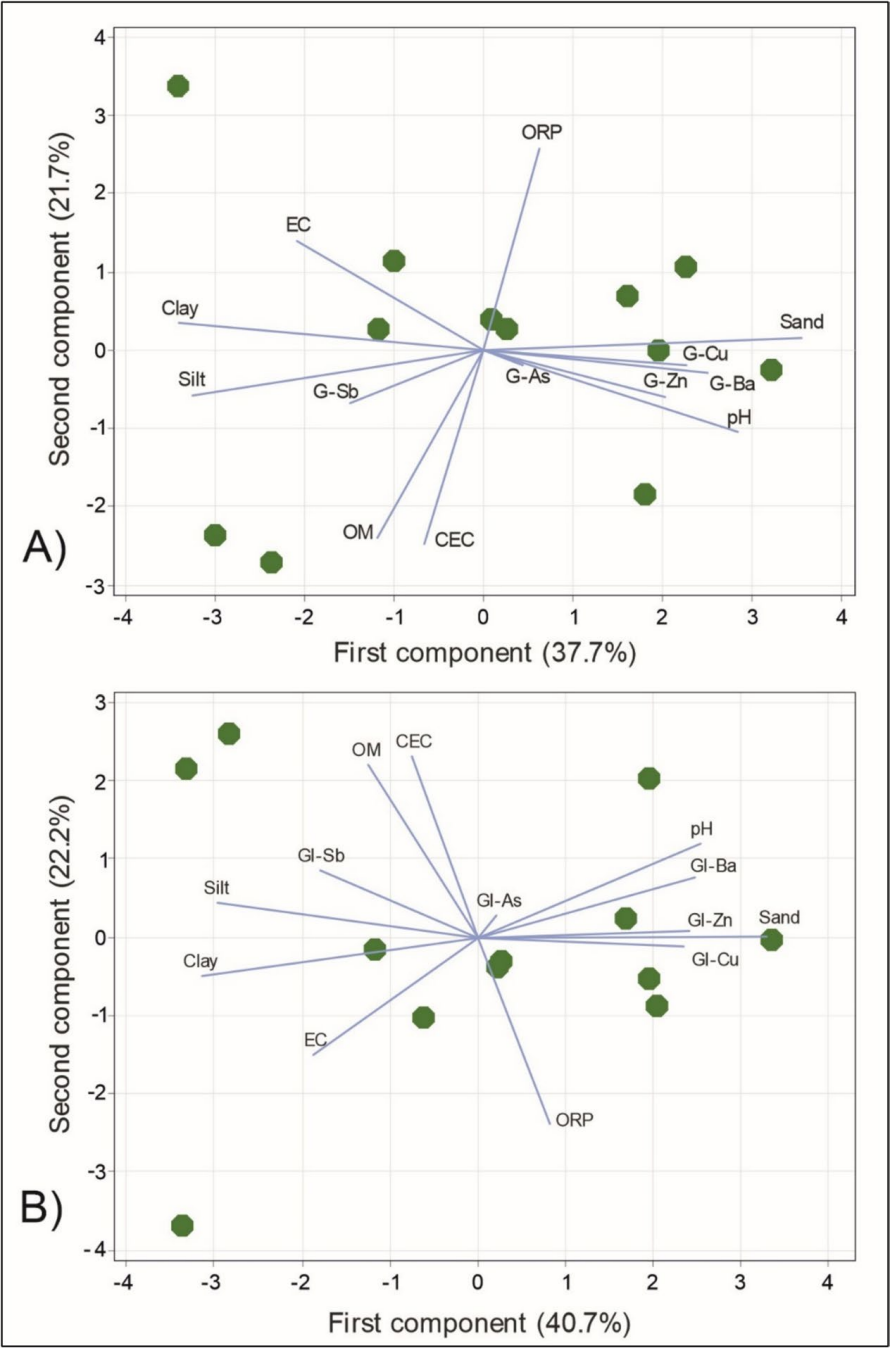
**a** Relationship between edaphic parameters and gastric bioaccessibility (G) of the PTEs, and **b** Relationship between edaphic parameters and gastrointestinal bioaccessibility (GI) of the PTEs

Table 2a and Fig. 5a shows the relationship between edaphic parameters and gastric bioaccessibility (G) of the PTEs in this study. Although the relationships were not so strong, the most significant in the first main component (PC1) and positively related are pH (0.346) with G-Ba (0.306), sand (0.433), G-Cu (0.276), and G -Zn (0.248), and negatively related with clay (− 0.414) and silt (− 0.396); while in the second principal component (PC2), ORP (0.535) were related with EC (0.289), and negatively related with OM (− 0.501) and CEC (− 0.517). In the third principal component (PC3), G-As (− 0.690), and G-Sb (− 0.565) were related.

**Table 2:** Principal component (PC) analysis matrix for the relationship between (a) gastric bioaccessibility and (b) gastrointestinal bioaccessibility of the PTEs and edaphic parameters

(a) Gastric bioaccessibility (G)				(b) Gastrointestinal bioaccessibility (GI)			
Variable	PC1	PC2	PC3	Variable	PC1	PC2	PC3
G-As	0.053	− 0.042	**− 0.690**	GI− As	0.026	0.062	**− 0.672**
G-Ba	**0.306**	− 0.062	0.258	GI− Ba	**0.311**	0.166	0.313
G-Cu	**0.276**	− 0.043	− 0.031	GI− Cu	**0.294**	− 0.025	− 0.110
G-Sb	− 0.182	− 0.141	**− 0.565**	GI− Sb	− 0.228	0.185	**− 0.531**
G-Zn	**0.248**	− 0.124	0.019	GI− Zn	**0.304**	0.019	− 0.122
pH	**0.346**	− 0.221	− 0.004	pH	**0.320**	**0.258**	0.018
EC	− 0.254	**0.289**	0.231	EC	− 0.236	**− 0.324**	0.227
ORP	0.077	**0.535**	0.066	ORP	0.103	**− 0.515**	0.072
OM	− 0.145	**− 0.501**	0.234	OM	− 0.159	**0.477**	0.242
CEC	− 0.081	**− 0.517**	0.131	CEC	− 0.095	**0.499**	0.144
Clay	**− 0.414**	0.070	− 0.003	Clay	**− 0.398**	− 0.106	0.001
Silt	**− 0.396**	− 0.121	0.069	Silt	**− 0.374**	0.095	0.064
Sand	**0.433**	0.031	− 0.037	Sand	**0.413**	0.002	− 0.036

Numbers in bold correspond to principal component more significative

Table 2b and Fig. 5b present the relationship between edaphic parameters and gastrointestinal bioaccessibility (GI). Likewise, the relationships were not so strong, the most significant in the first main component (PC1) and positively related parameters include pH (0.320) with GI-Ba (0.311), sand (0.413), GI-Zn (0.304), and GI-Cu (0.294), and negatively related with clay (− 0.398) and silt (− 0.374). In the second principal component (PC2), OM (0.477), CEC (0.499) and pH (0.258) were positively related, and negatively related with ORP (− 0.515) and EC (− 0.324). In the third principal component (PC3), GI-As (− 0.672), and G-Sb (− 0.531) were related.

The factorial analysis may indicate that the variables involved in factor 1 would be fundamentally the acid pH in the gastric phase, favoring the mobility of Zn, Cu and Ba, and linked to the sandy texture of the sediments; while the second factor would be constituted by physicochemical parameters that may influence the mobility of ETPs (EC and ORP), while OM and CEC may be involved in the immobilization of some of them; in the third factor are grouped the ETPs with similar chemical dispersion patterns, including the metalloids As and Sb. This relationship between the physicochemical parameters seems clear among themselves, but their influence on the evolution of the PTEs is not so clear. The distribution of variables among the 3 factors in the gastrointestinal phase is very similar, except for the importance of pH in the second factor (composed of the physicochemical parameters), where neutral pH, OM and CEC plays an important role in decreasing the bioaccessibility of the elements Ba, Cu and Zn (Bravo et al., 2017), and promoting the bioaccessibility of Sb (Bolan et al., 2022).

The geochemical composition of the sediments, and their grain size and texture influence the bioaccessibility of PTEs (Karna et al., 2017): sandy texture together with acid pH favors the bioaccessibility of the more mobile elements (Ba, Cu and Zn) in the gastric phase, while clay and silt have the opposite effect of making them less bioaccessible.

Studies show that metal concentrations tend to increase as particle size decreases. For example, Pb and Cd levels were observed to be three times higher in particles smaller than 0.3 μm as compared to those around 5 μm (Pelfrêne & Douay, 2018). This means that smaller particles tend to have higher bioaccessibility, because they dissolve more easily in gastric fluids. Research using the Unified BARGE Method (UBM) confirms that fine soil and dust particles contain more metals and release higher amounts of Cd and Pb during digestion than coarser particles (Pelfrêne & Douay, 2018). This is due to their larger surface area relative to their volume, which enhances metal adsorption and desorption. However, when comparing particles smaller than 250 μm with those smaller than 150 μm, there were no significant differences in the percentage of bioaccessible Pb and As (Karna et al., 2017).

These findings are important for managing sediments in recreational areas. Locations with more fine-grained sediments may pose a higher risk of metal exposure, especially in places where people, particularly children, frequently come into contact with the sediments, such as river bathing areas.

### Probabilistic risk assessment

**Fig. 6:**
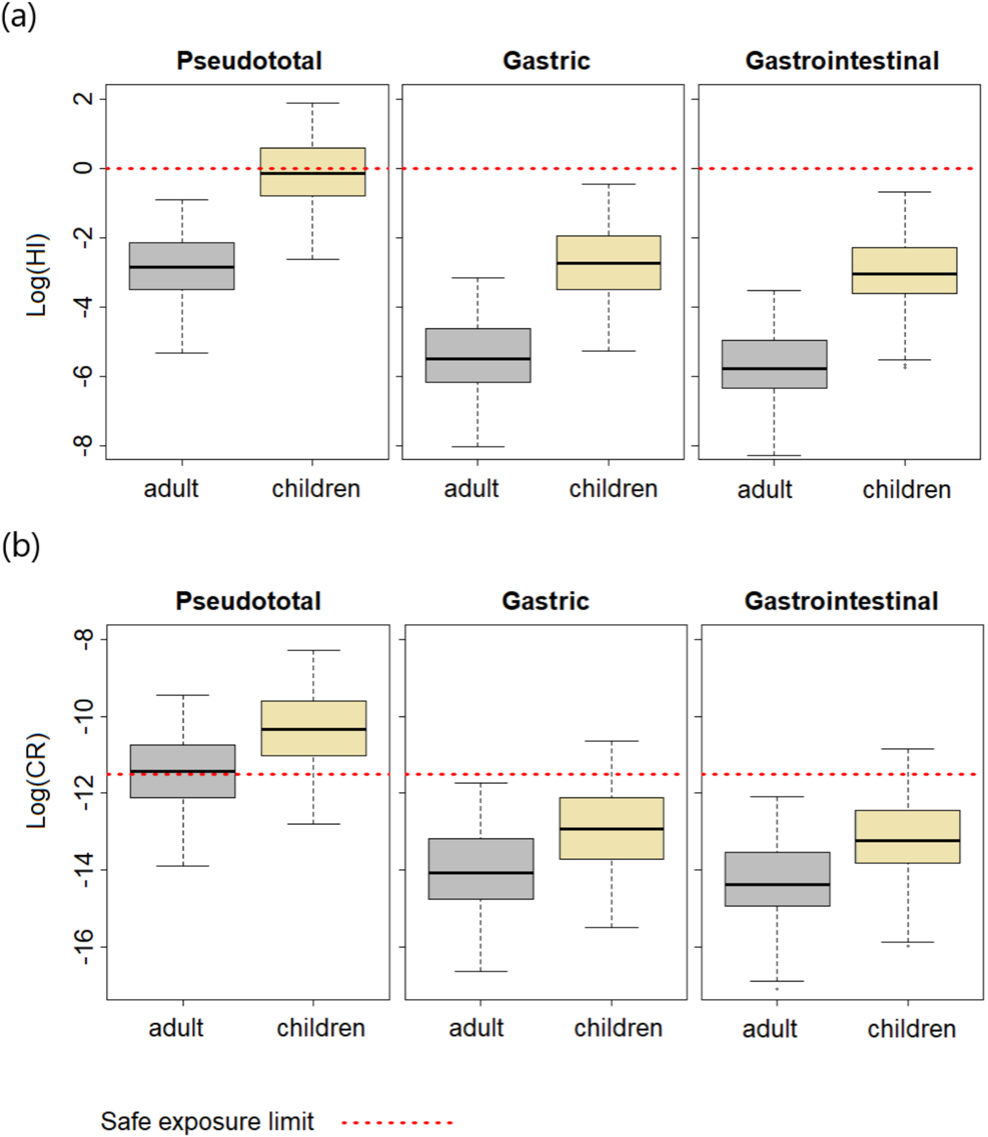
**a** Non-carcinogenic risk (HI) and **b** Carcinogenic risk (CR) for both receptors exposed to polluted stream sediments in the Remance mining area

Table S2 shows results of this study, including the probabilistic risk to human health estimated for pseudo-total concentrations, and for oral bioaccessibility in the gastric (G-phase) and gastrointestinal (GI-phase) phases. The non-cancer risk results (Fig. 6a) showed values that exceeded the safe exposure threshold only for the pseudo-total concentration analysis in children, at the 55th percentile: As was the contaminant that had the highest contribution to the risk outcomes. The HQ values for the rest of PTEs considered in this study were in the range between 1 × 10^–6^ and 1 × 10^–2^ for adults and between 1 × 10^–5^ and 1 × 10^–1^ in children, which corresponds to the pseudo-total concentration. For As, the HQ values in the G-phase and GI-phase were in the order of 10^–4^ and 10^–2^ for adults and 10^–3^ and 10^–1^ for children, which does not represent a risk. On the other hand, with respect to Ba, Cu, Sb, and Zn, for the G-phase the HQ varied in the order of 10^–7^ and 10^–4^ for adults and between 10^–6^ and 10^–3^ in children. For the GI-phase, the HQ values were in the range between 10^–8^ and 10^–4^ for adults and between 10^–7^ and 10^–3^ for children. These results show that the risk of developing adverse health effects is associated only with As in the pseudo-total concentration, since the risk values of the other elements are practically insignificant.

Reference doses (RfD) are a key factor in understanding why non-carcinogenic risks from metals such as Ba, Cu, Sb, and Zn are insignificant compared to As, despite their higher bioaccessibility. The reference dose is an estimate of the daily amount of a contaminant that is considered safe for long-term exposure without causing adverse health effects. For essential metals like Cu and Zn, reference doses are relatively high (RfDCu = 4.0 × 10^–2^ and RfDZn = 3.0 × 10^–1^) because the body needs these metals in small amounts for crucial biological functions and has homeostatic mechanisms to regulate their absorption (USDoE, 2024). This means that exposure to these metals, even at relatively higher levels in water or sediments, does not pose a significant health risk. In contrast, for As the reference dose is much lower (RfDAs = 3.0 × 10^–4^) due to its high toxicity, even at low concentrations (USDoE, 2024). Arsenic lacks effective regulatory mechanisms and accumulations in tissues, increasing its non-carcinogenic risk, especially over the long term.

Regarding the probabilistic carcinogenic risk (CR) for As exposure (Fig. 6b), this is exceeded for children in all fractions; in the pseudo-total concentration at the 10th percentile, so 90% of the recipients would be at risk; in the gastric phase at the 91st percentile, and therefore, 9% of recipients would be at risk; and for the gastrointestinal phase at the 95th percentile, and therefore, 5% of recipients would be at risk: The carcinogenic risk for children in the pseudo-total concentration was 10 times higher than in the gastric phase and 18 times higher than in the gastrointestinal phase. On the other hand, for adults the carcinogenic risk is exceeded only in the pseudo-total concentration at the 50th percentile, and so, 50% of the recipients would be at risk; but for the other fractions there is no risk.

The varying carcinogenic risk for As in different phases (pseudo-total, gastric, and gastrointestinal) suggests that its bioavailability changes depending on its chemical form and the digestive phase. The pseudo-total concentration represents the total amount of As in the sediment, including both bioavailable and non-bioavailable forms. Since the risk is exceeded at the 10th percentile in this phase for children, it indicates that a significant portion of As in the sediment is potentially accessible to organisms, especially during ingestion. In the gastric phase, where the As is exposed to acidic conditions like the stomach, the risk is lower, exceeding the threshold only at the 91st percentile. This suggests that, while some As is bioavailable in the stomach, the majority may be less soluble or less absorbed at this stage. In the gastrointestinal phase, where conditions resemble the small intestine, As is more likely to be absorbed into the bloodstream, explaining the higher carcinogenic risk (exceeding the threshold at the 95th percentile). This phase likely represents the greatest risk for bioavailability, as As becomes more soluble and available for absorption into the body. In general, the differences in carcinogenic risk across phases reflect the changing bioavailability of this element during digestion, with the pseudo-total phase representing total exposure and the gastric and gastrointestinal phases influencing the actual absorption and subsequent health impacts, particularly in children, whom are more vulnerable to these risks.

Although the accidental ingestion of stream sediments is an exposure route that is rarely considered in risk assessments, several studies report that this exposure route may pose a significant risk to exposed populations: Emenike et al. (2020), reported that incidental ingestion of polluted sediments is an important exposure route for residents, especially children in Nigeria, and (Jiménez-Oyola, Escobar Segovia, et al., 2021a, 2021b; Jiménez-Oyola et al., 2021a, 2021b) reported on the high carcinogenic and non-carcinogenic risk for children due to exposure to PETs in areas with mining influence in Ecuador.

Similar findings were reported in Italy (Mehta et al., 2020), Iran (Soltani et al., 2021) and the Czech Republic (Drahota et al., 2018), where the carcinogenic risk to human health was assessed from oral bioaccessibility test in contaminated waste and soils from abandoned mining areas, finding that As was the PTE with the highest contribution to the risk outcomes.

In agreement with the BAF values for As (2–17% in the G-phase and from 2 to 14% in the GI-phase), in our study the risk results were slightly higher in the G-phase than in the GI-phase for children.

Generally, it is evident that although As shows BAF values lower than 20% in both the G-phase and GI-phase, it is the PTE that poses the greatest risk to exposed populations, thus requiring a more thorough analysis. These results align with those reported by Das et al. (2013) and Wang et al. (2016). Although the human health risk in our study was assessed using bioaccessible concentrations (instead of traditional methods), which decreases the likelihood of underestimating exposure risk, further epidemiological and toxicological research is still needed to draw clear and accurate conclusions.

## Discussion

### Potential impacts of PTEs on human health

The Remance gold mine, located in the Veraguas province of Panama, has been exploited intermittently since the nineteenth century. As a result of mining activity, three abandoned tailings dams containing highly toxic mining waste have been exposed to the environment, causing the contamination of several environmental compartments, including stream sediments. This represents a potential risk to the health of the ecosystem and the population living in these contaminated environments.

The results obtained in this study reveal a concerning situation regarding the risks to human health associated with exposure to PTEs in stream sediments in the Remance area, especially for children participating in recreational activities in local rivers.

The probabilistic non-carcinogenic risk was exceeded only for children in the pseudo-total concentration at the 55th percentile, so 45% of recipients are at risk. As was the most significant contaminant, while Ba, Cu, Sb, and Zn, had a negligible contribution to risk outcomes.

Regarding the carcinogenic risk related to As-exposure, the results show that the safe exposure limit threshold is exceeded in all fractions for children, with 90%, 9%, and 5% of recipients at risk in the pseudo-total concentration, G-phase, and GI-phase, respectively. This indicates that even at relatively low levels of exposure, accidental ingestion of contaminated sediment could have serious adverse effects on their long-term health.

The difference between children and adults in terms of risk is notable, with children showing significantly greater vulnerability: while in adults the carcinogenic risk is exceeded only in the pseudo-total concentration at the 50th percentile, indicating that half of adult recipients are at risk, for children this risk extends to all the evaluated fractions.

The difference in carcinogenic risk between children and adults can have a significant impact on the formulation of public health policies and risk mitigation strategies in areas with contaminated river sediments. This approach considers the specific vulnerabilities of children, who are often more susceptible to the effects of toxic contaminants due to their physiology, behaviors, and stage of development (USEPA, 2014). Children, especially younger ones, tend to have direct contact with sediments through play. They are also more likely to put their hands in their mouths (Barbieri et al., 2014), which can increase the probability of ingesting contaminants present in the sediments. Since children are still growing and developing, early exposure to carcinogens can have long-term effects, with a higher risk of developing cancer later in life. Public health policies must take this factor into account, not only considering current exposures but also life-long risk projections. Public policies can be designed to identify and prioritize areas where children are most exposed to contaminants, such as parks, recreational areas, or residential areas near contaminated rivers. This could lead to greater investment in the remediation of these sites and the creation of protective barriers.

It is widely known that children are particularly vulnerable to the effects of As on neurological and cognitive development (Rahman et al., 2021). Chronic exposure during childhood can lead to learning problems, behavioral disorders, and developmental delays. Ingestion of As-contaminated sludge can cause irritation of the gastrointestinal tract, resulting in symptoms such as abdominal pain, diarrhea, and upset stomach (Fowler et al., 2014). Additionally, it can cause skin problems, such as pigmentation changes, skin lesions, and keratosis (Bini & Wahsha, 2014). Chronic exposure to As is associated with an increased risk of developing certain types of cancer in adults. Therefore, this metalloid is considered as a known carcinogen by the International Agency for Research on Cancer (IARC) and has been linked to several types of cancer in epidemiological and experimental studies (García-Pérez et al., 2019). Some of the types of cancer associated with As exposure include skin, lung-, bladder-, and kidney cancers (Martinez et al., 2011).

### Management and public policy: suggested actions

This study can serve as a starting point for toxicological investigations and the design of new strategies to assess potentially contaminated sites. PTEs can behave differently in different matrices, so each case needs to be investigated thoroughly. When formulating public policies, those responsible should consider bioaccessibility results as a fundamental criterion in the risk assessment of each affected area, also considering the geochemical behaviour of PTEs, to have adequate information to allow accurate estimates of the risk to human health based on the environmental matrix Boim et al., (2021).

The findings of this study highlight the urgent need to address pollution in stream sediments from mining areas in general, and in the Remance area in particular, implementing measures to reduce the exposure of the population, especially children, to these hazardous pollutants. Measures such as cleaning up contaminated sediment and implementing effective environmental management strategies are essential to protect public health and ensure a safe environment for recreational activities in the area. Risk communication is essential to reduce the exposure of potential recipients to contaminated environments; by minimizing exposure, risk levels can be kept within acceptable margins (Rodrigo et al., 2020; Wcisło et al., 2016). These policies should be integrated into mine closure regulations, making it mandatory to monitor sediment composition—along with other environmental compartments such as soil, air, and biota—to ensure remediation measures are completed or supplemented if necessary. Additionally, this requirement should be backed by a financial guarantee, which will only be returned to the mining company once the administration has fully assessed and verified compliance with all mandatory actions outlined in the mine closure plan.

The results of this study can support strategic judgments about the use of local rivers to reduce the risk to the inhabitants of the mining community. The probability of a person developing cancer over their lifetime caused by exposure to As through accidental ingestion of sediment was higher than acceptable levels, mainly for children. Therefore, due to exposure to As, the population should be monitored for early signs of As poisoning and the protection of vulnerable people living in the study area should be prioritized.

Given that children are a vulnerable population and that risk levels exceed safe exposure limits, recreational activities in the rivers of the study area are not recommended. In this sense, monitoring the quality of sediments and controlling the activities carried out in contaminated areas is crucial to preserve the health of river users.

To effectively monitor sediment quality in the study area and avoid further exposure to hazardous contaminants, it is crucial to implement a continuous, multidimensional monitoring system. First, strategic sampling points should be established in key areas, such as near potential sources of contamination and in areas where people may come into contact with sediments. Periodic analysis of TPE levels, such as As, should be conducted using advanced analytical techniques to ensure the accuracy of the results. In addition, alert thresholds should be set based on national and international regulations to identify dangerous levels of contamination. Along with physicochemical monitoring, biological assessments should be carried out to determine the effects of contaminants on local fauna and aquatic biodiversity. At the same time, educational programs should be implemented to raise community awareness of exposure risks and promote mitigation practices. Finally, local authorities should review and update environmental management policies and regulations, ensuring effective controls, such as the remediation of contaminated areas and the adoption of cleaner technologies to reduce pollution sources.

## Conclusions

This is the first study to assess the risk of exposure to contaminated sediments in a mining area in Panama, considering the oral bioaccessibility of PTEs (As, Ba, Cu, Sb and Zn). The probabilistic risk associated with the exposure of adults and children to these contaminants by accidental ingestion of sediments during recreational activities was estimated. The carcinogenic risk in children, calculated for the pseudo-total concentration, is 10–18 times higher than in the gastric and gastrointestinal phases, respectively. This reinforces the idea that results based on the pseudo-total concentration overestimate the risk, since they do not necessarily represent real exposure. In contrast, the evaluations made for the bioaccessible fractions, both gastric and gastrointestinal, offer a better estimate of the risk of exposure, since they consider the behavior of the contaminants in the body.

Unacceptable levels of non-carcinogenic and carcinogenic risk are due to the presence of As. Although this element has a low bioavailability (< 10%) in the gastric and gastrointestinal phases, the high concentration in the samples analyzed and its high toxicity make it a risk contaminant, mainly for children. The As in the sediments of the streams is anthropogenic in origin and has been dispersed from the tailings and mining activity areas to the streams through the runoff process.

To reduce human exposure to As in the Remance mining area, immediate action must be taken, including both preventive and remedial strategies. First, it is crucial to establish continuous monitoring of water and sediment quality in high-risk areas, aimed to track the spatial and temporal evolution of arsenic concentration and bioavailability. Ongoing sources of contamination, such as acid mine drainage and dispersal of material from tailings to rivers, should also be identified and decommissioned, as they may be contributing to the release of this element. In addition, human activities in areas with high As concentrations should be prohibited or restricted, especially in areas of direct exposure, such as contaminated rivers or agricultural areas, to reduce the contact with this contaminant.

It is paramount to integrate predictive models to assess how contaminants may move or accumulate over time, considering climatic factors and mining activity. Alert thresholds based on national and international standards should be established, along with dynamic data management, where monitoring results are accessible and updated in real time for local authorities, researchers and the community. In addition, to ensure the effectiveness of monitoring strategies, long-term health effects should be assessed through epidemiological studies in local populations and wildlife, using specific health indicators such as biomarker analysis of exposure. Finally, community participation in data collection and decision-making processes should be encouraged to ensure that measures taken are adaptable and relevant to local conditions.

Finally, we believe this study has provided valuable insights into managing contamination concerns in sediments. Although the research focuses on sediments from a mining area, the tools employed have proven effective in generating significant information with potential applications for other types of contaminated sediments associated with various human activities. In most cases, additional fundamental research will likely be required to identify the key factors driving local risks related to the presence of PTEs in this environmental compartment.

## Supplementary Information

Below is the link to the electronic supplementary material.

Supplementary file1 (DOCX 39 KB)

## Data Availability

No datasets were generated or analysed during the current study.
