# Supplementary material for: Oral bioaccessibility and probabilistic human health risk assessment of potentially toxic elements in stream sediments from an abandoned gold mine in Panama
Source: Environ Geochem Health. 2025 May 23;47(6):224. doi: 10.1007/s10653-025-02535-4 (PMC12102004; doi:10.1007/s10653-025-02535-4)
Supplement: Supplementary file 1 — Supplementary file1 (DOCX 39 KB) [file 10653_2025_2535_MOESM1_ESM.docx]

**Oral bioaccessibility and probabilistic human health risk assessment of potentially toxic elements in stream sediments from an abandoned gold mine in Panama**

**Ana González-Valoys^1,2,3*^, Samantha Jiménez-Oyola^4^, Carla Patinha^5^, Eva M^a^ García-Nogüero^6^, Jesús Peco^7^, Felipe Segundo**^1^**, José Ignacio Barquero^8^, Miguel Vargas-Lombardo^2,9^, José María Esbrí^10^, Pablo Higueras^8^**

^1^ Facultad de Ingeniería Civil, Universidad Tecnológica de Panamá, Ricardo J. Alfaro Avenue, Dr. Víctor Levi Sasso University Campus, Panamá City 0819-07289, Panama

^2^ SNI-SENACYT Sistema Nacional de Investigación-Secretaria Nacional de Ciencia, Tecnología e Innovación, Clayton, Ciudad del Saber Edif.205, Panama City 0816-02852, Panama

^3^ Centro de Estudios Multidisciplinarios en Ciencia, Ingeniería y Tecnología (CEMCIT-AIP), Ricardo J. Alfaro Avenue, Dr. Víctor Levi Sasso University Campus, Panamá City 0819-07289, Panama

^4^ Escuela Superior Politécnica del Litoral, ESPOL, Facultad de Ingeniería en Ciencias de la Tierra, Campus Gustavo Galindo km 30.5 vía Perimetral, P.O. Box 09-01-5863, Guayaquil, Ecuador.

^5^ GeoBioTec, GeoBioSciences, GeoTechnologies and GeoEngineering Research Center and Department of Geosciences, University of Aveiro, Campus de Santiago, 3810-193 Aveiro, Portugal

^6^Instituto de Educación Secundaria (IES) Mercurio, Almadén, 13400 Ciudad Real, Spain

^7^ Universidad Politécnica de Madrid, Madrid, Spain

^8^ Instituto de Geología Aplicada, Universidad de Castilla-La Mancha, EIMI Almadén, Plaza Manuel Meca 1, Almadén, 13400 Ciudad Real, Spain

^9^ Facultad de Ingeniería de Sistemas Computacionales, Universidad Tecnológica de Panamá, Ricardo J. Alfaro Avenue, Dr. Víctor Levi Sasso University Campus, Panamá City 0819-07289, Panama

^10^ Departamento de Mineralogía y Petrología, Universidad Complutense de Madrid, José Antonio Novais 12, 28040 Madrid, Spain

* Corresponding author [ana.gonzalez1@utp.ac.pa](mailto:ana.gonzalez1@utp.ac.pa)

**Table S1.** Statistical summary of pseudo-total contents of PTEs, and data on bioaccessible levels in gastric and gastro-intestinal phases as well as calculated bioaccessible fractions (BAF)

|  |  | Fractions | | | | | Bioaccessible fractions (BAF) | | | | |
| --- | --- | --- | --- | --- | --- | --- | --- | --- | --- | --- | --- |
|  |  | Cu | As | Zn | Sb | Ba | Cu | As | Zn | Sb | Ba |
|  |  | mg kg-1 | | | | | % | | | | |
| Pseudo-total concentrations | Min | 17,5 | 82,7 | 15,2 | 0,1 | 89,4 |  |  |  |  |  |
|  | p50 | 62,7 | 227,2 | 61,7 | 4,9 | 208,1 |  |  |  |  |  |
|  | p95 | 115,8 | 703,8 | 171,7 | 25,6 | 483,8 |  |  |  |  |  |
|  | max | 119,1 | 784,1 | 203,6 | 25,7 | 613,8 |  |  |  |  |  |
|  | SD | 38,4 | 227,5 | 53,9 | 9,4 | 145,2 |  |  |  |  |  |
|  | LQ | 0,2 | 0,5 | 2,0 | 0,2 | 0,5 |  |  |  |  |  |
|  |  |  |  |  |  |  |  |  |  |  |  |
| Oral bioaccessibility  (Gastric phase –  UBM method) | Min | 1,6 | 4,0 | 5,5 | < 0.05 | 1,2 | 9,4 | 1,5 | 10,6 | 0,3 | 0,4 |
|  | p50 | 10,5 | 19,0 | 10,2 | 0,1 | 28,2 | 22,7 | 8,8 | 18,7 | 1,7 | 19,5 |
|  | p95 | 37,6 | 67,2 | 31,5 | 0,3 | 124,5 | 41,1 | 16,4 | 39,3 | 25,5 | 69,3 |
|  | max | 41,8 | 67,8 | 46,6 | 0,4 | 126,3 | 42,6 | 17,2 | 43,5 | 47,7 | 78,6 |
|  | SD | 12,7 | 22,1 | 11,0 | 0,1 | 49,4 | 11,4 | 5,2 | 10,4 | 13,9 | 26,7 |
|  | LQ | 0,1 | 0,1 | 0,5 | 0,05 | 0,2 |  |  |  |  |  |
|  |  |  |  |  |  |  |  |  |  |  |  |
| Oral bioaccessibility  (Gastrointestinal phase –  UBM method) | Min | 2,5 | 3,6 | 4,9 | 0,1 | 0,2 | 9,6 | 1,7 | 7,5 | 0,5 | 0,1 |
|  | p50 | 7,6 | 11,5 | 8,7 | 0,1 | 15,4 | 16,9 | 7,5 | 12,8 | 2,7 | 9,5 |
|  | p95 | 29,0 | 56,1 | 14,9 | 0,5 | 59,2 | 31,4 | 13,2 | 49,0 | 51,7 | 31,3 |
|  | max | 31,8 | 58,6 | 18,8 | 0,6 | 67,1 | 32,5 | 13,6 | 70,7 | 89,3 | 31,9 |
|  | SD | 9,6 | 18,8 | 3,9 | 0,2 | 22,5 | 8,0 | 4,3 | 18,2 | 27,5 | 11,3 |
|  | LQ | 0,1 | 0,1 | 0,5 | 0,05 | 0,2 |  |  |  |  |  |

Notes: SD, standard deviation; LQ, limit of quantification.

**Table S2.** Statistical summary for probabilistic non-carcinogenic (HQ) and carcinogenic risk (CR). Numbers in bold correspond to values that exceed the safe exposure limit for HQ (1) and CR (1E-05).

| **Fractions** |  | **As** | | | | **Ba** | | **Cu** | | **Sb** | | **Zn** | |
| --- | --- | --- | --- | --- | --- | --- | --- | --- | --- | --- | --- | --- | --- |
|  |  | **HQ_a** | **HQ_c** | **CR_a** | **CR_c** | **HQ_a** | **HQ_c** | **HQ_a** | **HQ_c** | **HQ_a** | **HQ_c** | **HQ_a** | **HQ_c** |
| **Pseudo-total concentrations** | Min | 4.78E-03 | 7.18E-02 | 9.21E-07 | 2.77E-06 | 8.96E-06 | 9.89E-05 | 9.57E-06 | 1.46E-04 | 5.72E-06 | 8.30E-05 | 1.16E-06 | 1.48E-05 |
|  | p50 | 5.62E-02 | 8.42E-01 | **1.08E-05** | **3.25E-05** | 7.86E-05 | 1.15E-03 | 1.01E-04 | 1.51E-03 | 9.71E-04 | 1.46E-02 | 1.44E-05 | 2.16E-04 |
|  | p95 | 2.34E-01 | **3.61E+00** | **4.52E-05** | **1.39E-04** | 2.21E-04 | 3.59E-03 | 3.04E-04 | 4.63E-03 | 6.34E-03 | 9.25E-02 | 5.14E-05 | 8.07E-04 |
|  | max | 4.02E-01 | **6.46E+00** | **7.74E-05** | **2.49E-04** | 5.51E-04 | 7.83E-03 | 4.39E-04 | 7.59E-03 | 1.14E-02 | 1.61E-01 | 1.23E-04 | 1.92E-03 |
|  | SD | 7.19E-02 | 1.12E+00 | 1.39E-05 | 4.34E-05 | 6.72E-05 | 1.06E-03 | 8.93E-05 | 1.39E-03 | 2.07E-03 | 3.17E-02 | 1.61E-05 | 2.54E-04 |
| **Oral bioaccessibility**  **(Gastric phase –**  **UBM method)** | Min | 3.13E-04 | 4.82E-03 | 6.04E-08 | 1.86E-07 | 1.13E-07 | 1.21E-06 | 9.75E-07 | 1.43E-05 | 2.69E-06 | 2.90E-05 | 4.16E-07 | 5.44E-06 |
|  | p50 | 4.02E-03 | 6.30E-02 | 7.76E-07 | 2.43E-06 | 1.15E-05 | 1.66E-04 | 1.88E-05 | 2.80E-04 | 1.67E-05 | 2.50E-04 | 2.52E-06 | 3.82E-05 |
|  | p95 | 2.15E-02 | 3.34E-01 | 4.14E-06 | **1.29E-05** | 6.68E-05 | 1.03E-03 | 8.90E-05 | 1.34E-03 | 7.24E-05 | 1.11E-03 | 1.14E-05 | 1.76E-04 |
|  | max | 4.20E-02 | 6.23E-01 | 8.10E-06 | **2.40E-05** | 1.06E-04 | 1.90E-03 | 1.97E-04 | 2.63E-03 | 1.83E-04 | 2.83E-03 | 2.18E-05 | 4.26E-04 |
|  | SD | 6.84E-03 | 1.06E-01 | 1.32E-06 | 4.09E-06 | 2.20E-05 | 3.43E-04 | 2.71E-05 | 4.27E-04 | 2.31E-05 | 3.68E-04 | 3.31E-06 | 5.25E-05 |
| **Oral bioaccessibility**  **(Gastrointestinal phase –**  **UBM method)** | Min | 1.98E-04 | 2.98E-03 | 3.82E-08 | 1.15E-07 | 2.61E-08 | 2.89E-07 | 1.41E-06 | 2.11E-05 | 3.33E-06 | 4.46E-05 | 3.16E-07 | 4.33E-06 |
|  | p50 | 3.01E-03 | 4.62E-02 | 5.80E-07 | 1.78E-06 | 7.06E-06 | 1.08E-04 | 1.62E-05 | 2.51E-04 | 2.33E-05 | 3.53E-04 | 2.02E-06 | 2.97E-05 |
|  | p95 | 1.71E-02 | 2.63E-01 | 3.29E-06 | **1.01E-05** | 2.96E-05 | 4.58E-04 | 7.19E-05 | 1.04E-03 | 1.16E-04 | 1.74E-03 | 5.06E-06 | 7.97E-05 |
|  | max | 2.94E-02 | 5.00E-01 | 5.67E-06 | **1.93E-05** | 6.10E-05 | 8.77E-04 | 1.17E-04 | 2.04E-03 | 2.11E-04 | 5.03E-03 | 1.10E-05 | 1.66E-04 |
|  | SD | 5.26E-03 | 8.43E-02 | 1.01E-06 | 3.25E-06 | 9.98E-06 | 1.54E-04 | 2.12E-05 | 3.31E-04 | 3.44E-05 | 5.36E-04 | 1.40E-06 | 2.28E-05 |

*a: adult; c: children*
